# Sustainable manganese catalysis for late-stage C–H functionalization of bioactive structural motifs

**DOI:** 10.3762/bjoc.17.122

**Published:** 2021-07-26

**Authors:** Jongwoo Son

**Affiliations:** 1 Department of Chemistry, Dong-A University, Busan 49315, South Korea; 2 Department of Chemical Engineering (BK21 FOUR Graduate Program), Dong-A University, Busan 49315, South Korea

**Keywords:** bioactive molecules, 3d transition metals, late-stage functionalization, manganese catalyst, sustainable catalysis

## Abstract

The late-stage C–H functionalization of bioactive structural motifs is a powerful synthetic strategy for accessing advanced agrochemicals, bioimaging materials, and drug candidates, among other complex molecules. While traditional late-stage diversification relies on the use of precious transition metals, the utilization of 3d transition metals is an emerging approach in organic synthesis. Among the 3d metals, manganese catalysts have gained increasing attention for late-stage diversification due to the sustainability, cost-effectiveness, ease of operation, and reduced toxicity. Herein, we summarize recent manganese-catalyzed late-stage C–H functionalization reactions of biologically active small molecules and complex peptides.

## Introduction

Manganese, a 3d transition metal, allows for a potentially ideal sustainable catalytic system because of the natural abundance, cost-effectiveness, and low toxicity. In addition, it presents variable oxidation states (−3 to +7), which enable diverse catalytically active manganese complexes, providing characteristic reaction profiles. Since the first pioneering manganese-mediated reaction for accessing azobenzenes was unveiled [1], manganese catalysts have exhibited a significant capacity for powerful C–H functionalization, and they have therefore been actively utilized in the area of sustainable organic syntheses [2–6].

Catalytic late-stage C–H functionalization, a highly efficient synthetic strategy, is regarded as a crucial tactic in the area of natural products, drug discovery, and medicinal chemistry [7–12] as it confers an invaluable synthetic opportunity for the facile diversification of biologically active complex molecules at the late stage. In recent years, much effort has been devoted to developing sustainable catalytic late-stage C–H functionalization methods that utilize naturally abundant 3d metal catalysts.

This review will provide an overview on recent studies on Mn-catalyzed late-stage C–H functionalization of challenging substrates, such as biologically active molecules and complex peptides, which are of great importance to medicinal chemists and are categorized according to the transformations involved.

## Review

### Manganese-catalyzed late-stage C–H fluorination

**Scheme 1:**
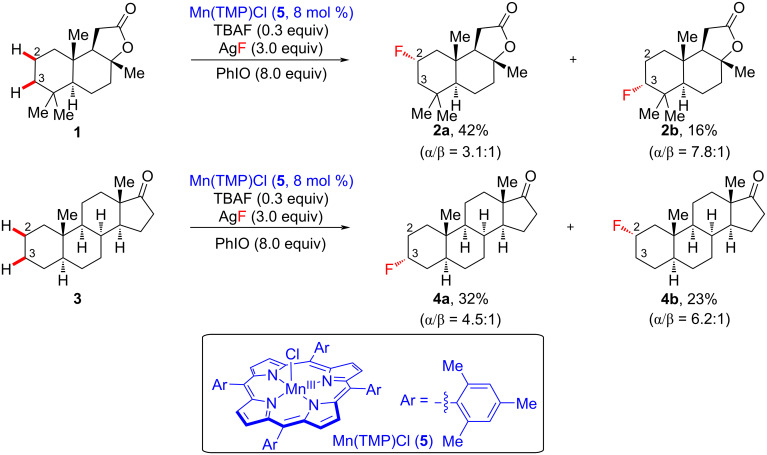
Mn-catalyzed late-stage fluorination of sclareolide (**1**) and complex steroid **3**.

The fluorination of organic molecules [13–16], a highly valuable synthetic transformation, has been widely investigated in medicinal chemistry and in the pharmaceutical industry as it generally imparts the targeted fluorinated molecules with dramatically improved physical, biological, and/or chemical properties [17–21]. Among the catalytic C–F forming processes, aliphatic late-stage fluorination (C_sp3_–F) is relatively challenging due to the omnipresent unactivated C–H bonds of the substrate molecules. In 2012, Groves et al. revealed a manganese porphyrin-catalyzed late-stage C_sp3_–H fluorination method (Scheme 1) [22]. In the authors’ approach, a direct late-stage process facilitated the fluorination of sclareolide (**1**) and complex steroid **3**. Sclareolide (**1**) is a naturally available terpenoid with antifungal and anticancer activities [23]. Under the optimized reaction conditions, sclareolide (**1**) is fluorinated at the C2 and C3 positions in 42% (see **2a**) and 16% yield (see **2b**), respectively. Therein, C2 fluorination was favored, and **2a** was observed as the major product due to the sterically congested environment at C3 created by the adjacent *gem*-dimethyl groups. The regioselectivity at the C2 position was observed similarly in a study on Mn-catalyzed chlorination [24]. This manganese porphyrin catalytic system was also effective in the direct site-selective fluorination of complex steroid scaffold **3**, containing 28 unactivated C–H bonds. Based on the authors’ analysis of steric and electronic factors, it was suggested that the methylene units at C2 and C3 of the A-ring system were the most reactive sites for hydrogen abstraction, yielding fluorinated steroids **4a** and **4b** in 32% and 23% yield, respectively. For both products, α-fluorination was dominant over β-fluorination, likely because α-fluorination occurred on the less sterically hindered face. Additionally, difluorination was observed with negligible amounts because the monofluorinated product is rendered more electronically deficient by the first fluorine atom.

**Figure 1:**
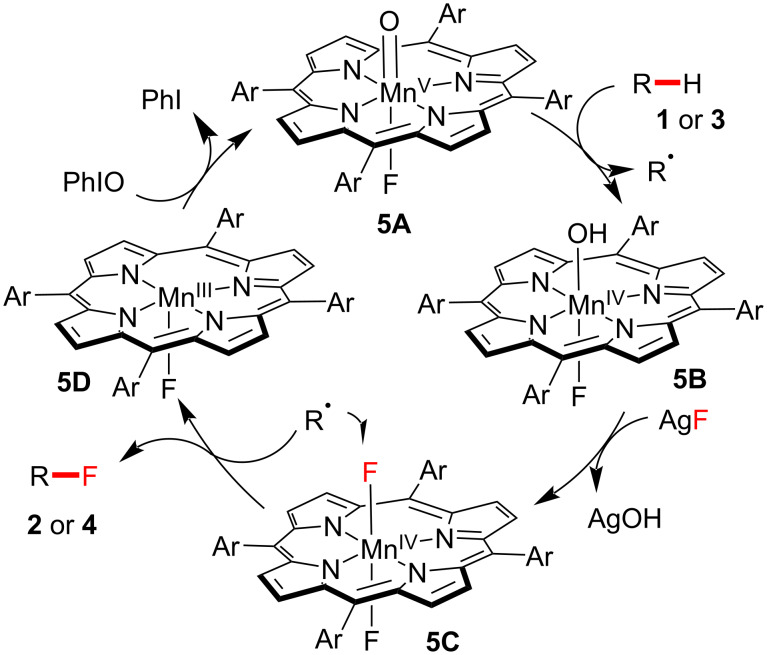
Proposed reaction mechanism of C–H fluorination by a manganese porphyrin catalyst.

Based on an analysis by DFT calculations, the postulated reaction pathway of manganese-catalyzed C–H fluorination is described in Figure 1. Initially, resting Mn(TMP)F undergoes oxidation, generating oxomanganese(V) complex O=Mn(TMP)F (**5A**), followed by H-abstraction of the substrate **1** or **3**, providing HO–Mn(TMP)F (**5B**) and a C-centered radical. The *trans*-difluoro-substituted Mn(TMP) intermediate **5C**, generated by an excess of the fluoride source, traps the C-centered radical, finally delivering the fluorinated product **2** or **4**.

**Scheme 2:**
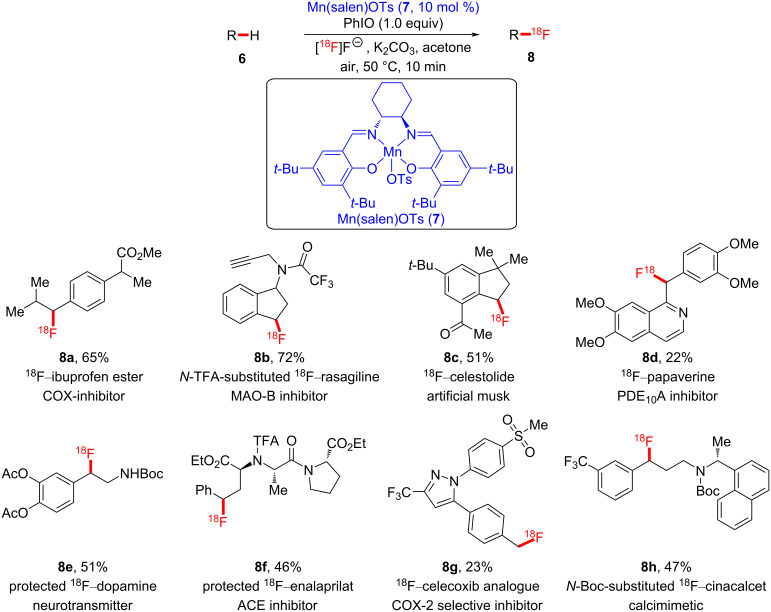
Late-stage radiofluorination of biologically active complex molecules.

Thereafter, the same group successively reported the first manganese-catalyzed late-stage ^18^F-fluorination of a wide range of biologically active compounds (Scheme 2) [25]. It is well known that the most utilized radioisotope for positron emission tomography (PET) in clinical and preclinical research is ^18^F. Radiopharmaceuticals should be prepared at the late stage of the entire synthetic protocol because of the short half-lives of radioisotopes [26–29]. In their study, the authors used an aqueous ^18^F-fluoride solution obtained by the nuclear reaction using a cyclotron, and manganese–salen complex **7** was used as a fluoride transfer catalyst, which facilitated late-stage C–H radiofluorination, affording the corresponding radiofluorinated bioactive molecules **8a**–**h**. In general, the regioselectivity of fluorination was observed at the less sterically hindered benzylic position.

**Figure 2:**
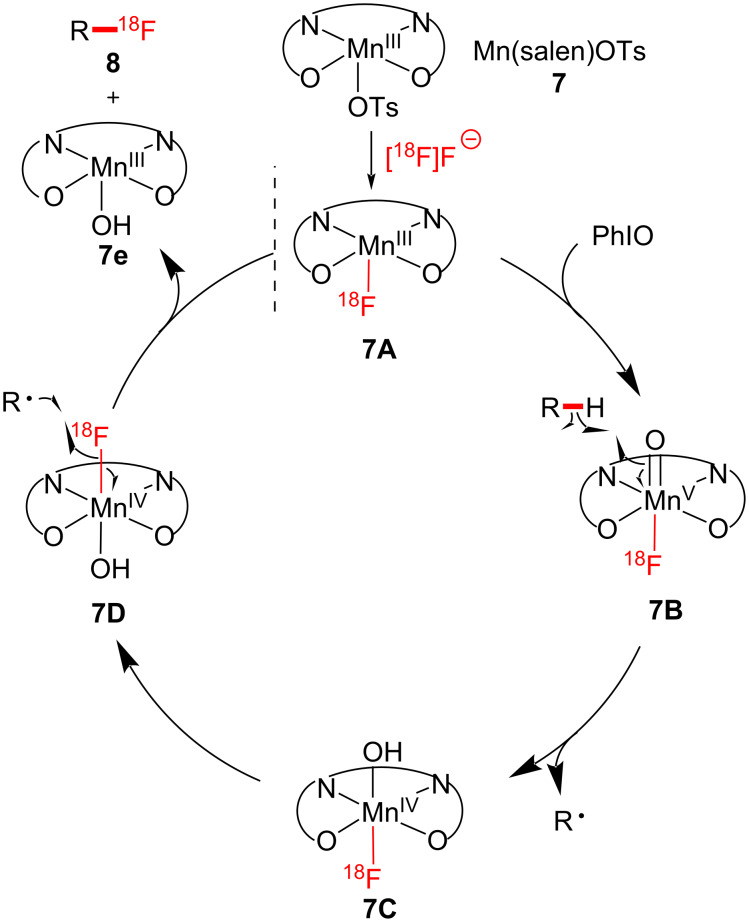
Proposed mechanism of C–H radiofluorination.

The proposed reaction mechanism for radiofluorination is depicted in Figure 2. Although the *trans*-difluoro-substituted Mn(IV) complex is the reactive F-transfer intermediate in ^19^F chemistry, the formation of a *trans*-^18^F-difluoro-substituted Mn(IV) complex is not reasonable because of the limiting amount of the ^18^F source. Therefore, it was suggested that ^18^F-fluorination is more likely to occur through an intermediate HO–Mn–^18^F motif as in **7C**. These manganese-catalyzed late-stage C–H fluorinations showcase a convenient and operationally simple fluorination strategy suitable for bioactive structural motifs.

### Manganese-catalyzed late-stage C–H azidation

In organic synthesis, organic azides are of considerable significance in the fields of medicinal chemistry, chemical biology, and nanotechnology as they can participate in elegant conjugative transformations, such as azide–alkyne [3 + 2]-cycloaddition [30–37].

**Scheme 3:**
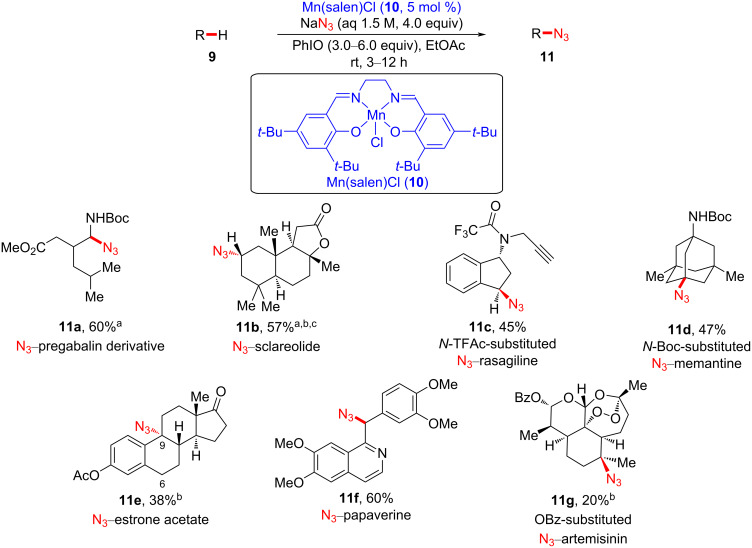
Late-stage C–H azidation of bioactive molecules. ^a^1.5 mol % of Mn(TMP)Cl (**5**) was used. ^b^Methyl acetate was used as a solvent. ^c^The ratio of α/β azidation was 7.5:1.

Based on their previous late-stage fluorination studies [22,25], Groves et al. further showcased a manganese(III)–salen-catalyzed azidation process using an aqueous azide solution as a convenient azide source to execute facile C–H azidation of pharmaceutical-like complex molecules (Scheme 3) [38]. In this study, the regioselectivity is governed not only by electronic and steric effects of the manganese catalysts **5** and **10** but also by the electronic properties of the substrates. Pregabalin is an anticonvulsant drug used to treat epilepsy and anxiety disorders [39], and an analogue of pregabalin was transformed to azidated derivative **11a**. It is noteworthy that positional selectivity was observed for the α-position of the carbamate functional group due to the stabilization of the carbon radical by the adjacent carbamate group. The naturally available substrate **9b** was shown to undergo the azidation process at the less sterically hindered position. An analogue of rasagiline (Azilect^®^), a Parkinson’s disease drug, successfully underwent benzylic azidation, providing product **11c**. Other complex molecules bearing aromatic groups were also successfully azidated, predominantly at the benzylic position (see **11e** and **11f**). Notably, diazidation of **9e** was observed as the major side reaction (18%). Interestingly, OBz-substituted artemisinin **9g** was converted to OBz-substituted N_3_–artemisinin **11g** via azidation at the tertiary position using the manganese–salen catalyst. Furthermore, this Mn-catalyzed azidation protocol is highly robust in air, highlighting the practical simplicity of late-stage C–H azidation of bioactive molecules.

**Figure 3:**
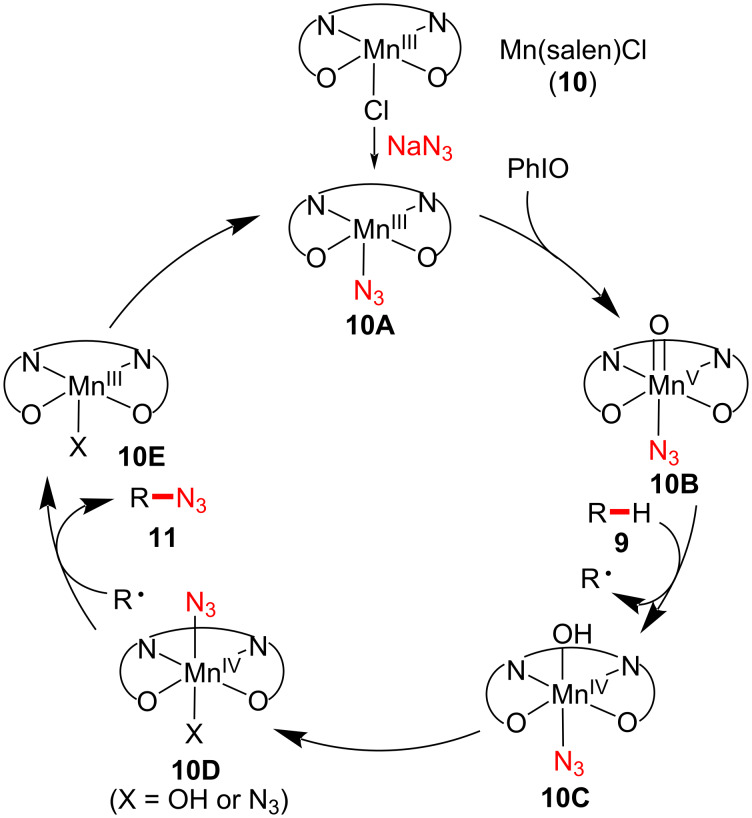
Proposed reaction mechanism of manganese-catalyzed C–H azidation.

A plausible reaction pathway was proposed, as illustrated in Figure 3. Similar to manganese-catalyzed C–H fluorination [22], the resting Mn(III) catalyst is oxidized to O=Mn(V)–N_3_ complex **10B**. Subsequently, an alkyl radical is generated upon H-abstraction by forming Mn(VI) intermediate **10C**. The resulting alkyl radical is then trapped by Mn(IV)–N_3_ intermediate **10D**, affording azidation product **11**. Upon regeneration, the catalyst participates in the next catalytic cycle. In this Mn-catalyzed azidation study, the azide/oxygenated product ratio was 2:1–4:1. Therefore, a chemoselective manner is of dire need to avoid unwanted C–H oxygenation.

**Scheme 4:**
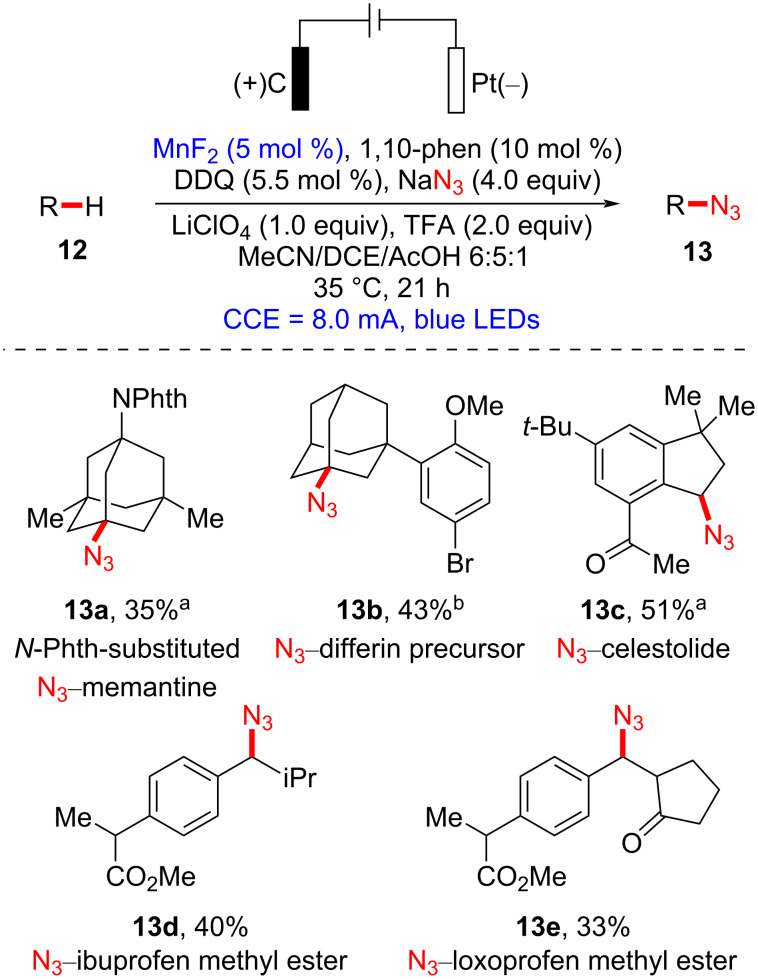
Mn-catalyzed late-stage C–H azidation of bioactive molecules via electrophotocatalysis. ^a^2.5 mol % of MnF_2_ was used. ^b^NaN_3_ (5.0 equiv), MnF_2_ (10 mol %), 1,10-phenanthroline (20 mol %), TFA (4.0 equiv), and LiClO_4_ (2.0 equiv) were used with 4.5 mA for 15 h (5.0 mmol scale).

In 2020, the Lei group disclosed the combination of a manganese catalyst and a electrophotocatalyst for the late-stage C_sp3_–H azidation of biologically active molecules in a selective and sustainable manner (Scheme 4) [40]. Memantine is a drug used to treat neurodegenerative disorders such as Alzheimer’s disease [41–42]. In the authors’ study, azidated *N*-protected memantine **13a** was successfully generated by employing electricity and visible-light irradiation in the presence of a Mn catalyst. High regioselectivity was observed at tertiary or benzylic positions (see **13a**–**c**). For commercially available drug derivatives, methyl esters of ibuprofen and loxoprofen underwent regioselective azidation at the secondary benzylic sites over tertiary benzylic sites (see **13d** and **13e**).

**Figure 4:**
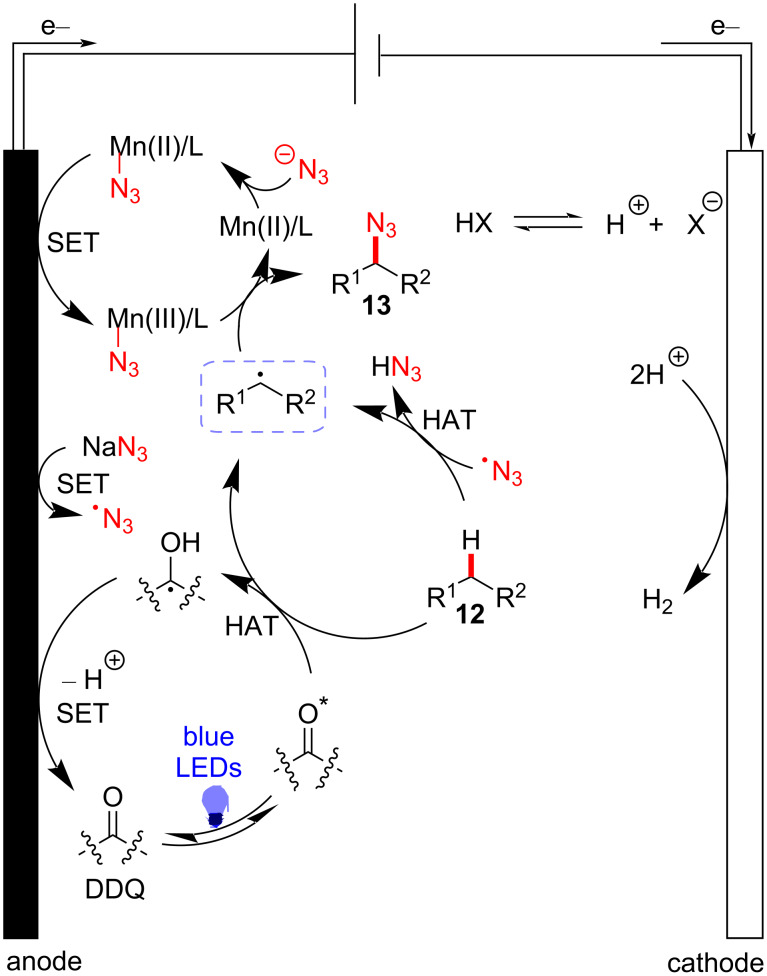
Proposed reaction mechanism of electrophotocatalytic azidation.

Additional mechanistic studies support the reaction pathway depicted in Figure 4. The azide anion is oxidized to a radical species on the anodic surface, where Mn(II)/L–N_3_ is also oxidized to Mn(III)/L–N_3_. Azide radical addition to Mn(II)/L to form Mn(III)/L–N_3_ was considered as a possible route. Concurrently, the photocatalyst is irradiated by blue LED light to induce hydrogen atom transfer (HAT) at the C–H bond of substrate **12**, generating alkyl radicals and enabling C–N_3_ bond formation to afford **13** via the reaction with Mn(III)/L–N_3_. The anodic surface oxidizes the radical adjacent to the hydroxy group of the photocatalyst, thereby regenerating it. At the same time, the hydrogen atom abstraction of radical species of photocatalyst by Mn(III)–N_3_ could not be excluded. This late-stage process by Mn catalysis, electrochemistry, and visible-light catalysis exhibits high value as a sustainable tool to investigate problematic synthetic transformations.

**Scheme 5:**
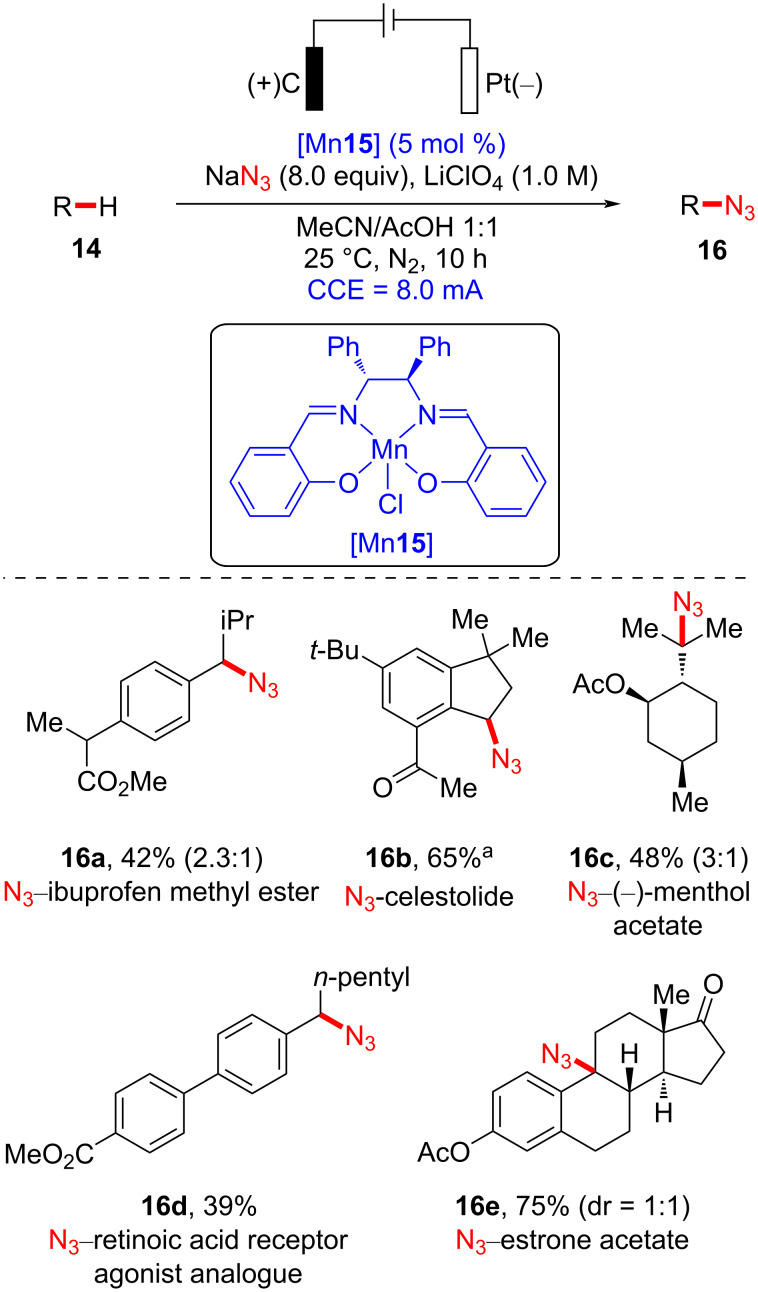
Manganaelectro-catalyzed late-stage azidation of bioactive molecules.

Recently, the Ackermann group disclosed a convenient manganese-catalyzed late-stage C–H azidation of bioactive molecules bearing unactivated C_sp3_–H bonds facilitated by electricity (Scheme 5) [43]. Several pharmaceutically active molecules were committed to the external oxidant-free reaction conditions and were shown to undergo chemoselective azidation. Azidation of ibuprofen methyl ester (**14a**) was selective for the secondary benzylic position over the tertiary. Similar regioselectivity pattern of ibuprofen methyl ester (**14a**) and celestolide (**14b**) was observed in a study on manganese-catalyzed isocyanation [44]. Moreover, (−)-menthol acetate (**14c**) was successfully azidated, indicating that azidation favors tertiary C–H bonds in less sterically congested environments. A mixture of diastereomers (1:1 ratio) resulted from the azidation of estrone acetate (**14e**), strongly supporting a radical reaction pathway.

**Figure 5:**
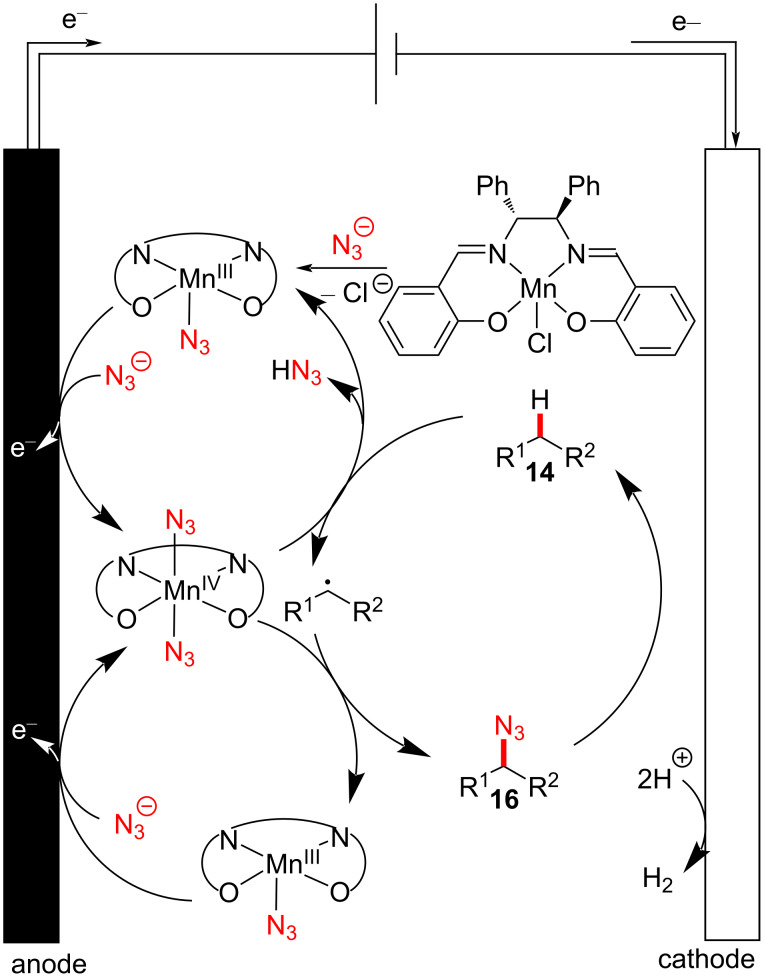
Proposed reaction pathway of manganaelectro-catalyzed late-stage C–H azidation.

Based on their additional mechanistic experiments, the authors suggested that the oxidation of the Mn(III) species to Mn(IV) takes place on the anodic surface, resulting in the formation of a *trans*-diazide Mn(IV) intermediate (Figure 5). The high-valent manganese(IV) complex is susceptible to HAT from the substrate **14**, generating an alkyl radical [45–46]. Subsequently, further azide radical transfer from the *trans*-diazide Mn(IV) complex was proposed to furnish the formation of the C–N_3_ bond.

### Manganese-catalyzed late-stage C–H amination

The installation of amine functional groups onto biologically active molecules is regarded as a potentially versatile synthetic transformation for accessing diverse potent candidates with tailored physical and biological properties [47]. For example, ampicillin, an analogue of benzylpenicillin (penicillin G), also used as an antibiotic, contains an amine group at the benzylic position [48]. Likewise, other commercially available small-molecule drugs, such as Plavix^®^ (antiplatelet), Gleevec^®^ (anticancer), and augmentin (antibiotic), also contain the benzylic amine motif. Therefore, C–H amination is synthetically important for the diversification of biologically active molecules. Transition metal catalysis has set the stage for C–H amination processes in organic syntheses [49]. To date, there are several examples of late-stage C–H amination methods that utilize iron and manganese as 3d transition metal catalysts [50–52]. However, intermolecular benzylic C–H amination has rarely been explored due to the challenges associated with selectivity and reactivity.

**Scheme 6:**
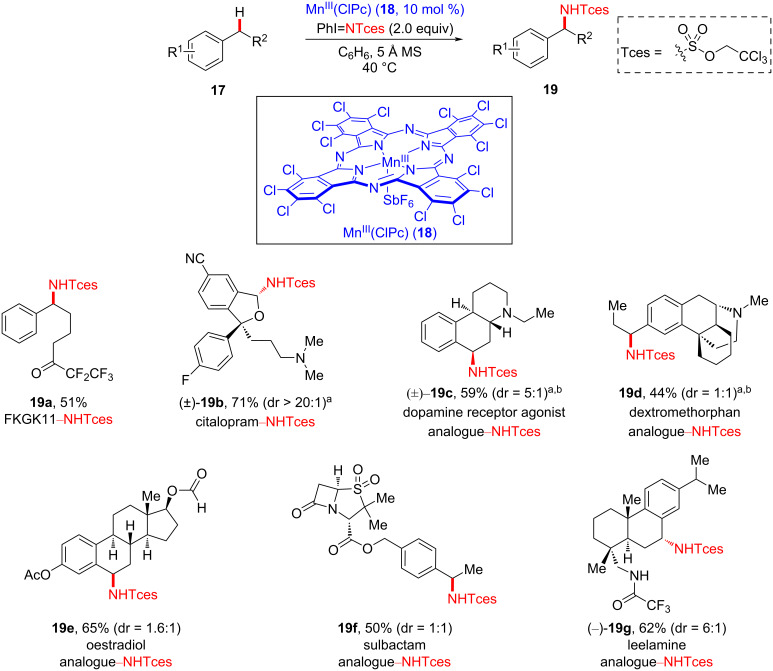
Mn-catalyzed late-stage amination of bioactive molecules. ^a^3 Å MS were used. Protonation with HBF_4_⋅OEt_2_ (1.1 equiv) in dichloromethane before amination, then deprotonation with 1 M NaOH in dichloromethane after amination. ^b^3.0 equiv of PhI=NTces were used.

In 2018, White et al. disclosed the late-stage manganese-catalyzed benzylic C–H amination of sophisticated biologically active molecules (Scheme 6) [53]. Their site-selective late-stage C–H amination strategy is practically scalable and convenient and exhibits excellent functional group tolerance. Several derivatives of complex biologically active compounds and natural products were evaluated in the late-stage benzylic C–H amination process using Mn^III^(ClPc) (**18**) and iminoiodinane. For example, the amination of FKGK11 (**17a**), a potent inhibitor of iPLA2, proceeded smoothly in a moderate yield. Notably, citalopram (**17b**), an antidepressant, was also reacted under amination conditions involving HBF_4_, affording product **19b** in good yield and excellent diastereoselectivity (dr > 20:1). Other multiple benzylic C–H bond-containing molecules, including a dopamine receptor agonist analogue **17c** as well as derivatives of dextromethorphan, oestradiol, sulbactam, and leelamine **17d**–**g**, respectively, were shown to undergo amination at the less sterically congested benzylic position, affording aminated products **19c**–**g**. Further traceless removal of the Tces group was also investigated under Zn/Cu coupling conditions to install the free NH functionality. These findings highlight the convenience of manganese catalysis for the late-stage benzylic C–H amination of sophisticated bioactive molecules and natural products.

**Figure 6:**
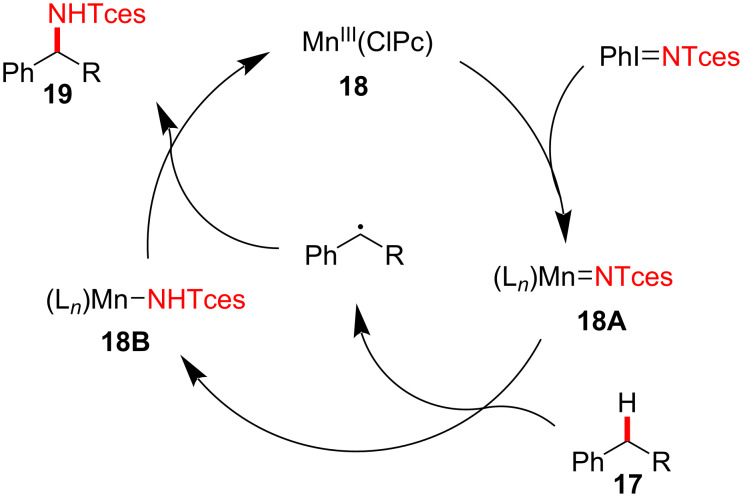
Proposed mechanism of manganese-catalyzed C–H amination.

The proposed mechanism of the Mn-catalyzed benzylic amination is shown in Figure 6. Initially, metallonitrene intermediate **18A** is formed from the reaction of catalyst **18** with iminoiodinane, which is subsequently transformed to Mn–imido complex **18B** via conversion of substrate **17** into a temporary benzylic radical species, wherein C–H bond cleavage is proposed to be the rate-determining step (inter- and intramolecular KIEs of C–H cleavage are 2.5 and 3.0, respectively). Next, the benzylic radical is trapped by the Mn–imido complex to afford aminated product **19**. Based on additional mechanistic experiments, it was suggested that the Mn^III^(ClPc) (**18**)-catalyzed C–H amination process is regioselective for the more electron-rich benzylic position, rationalizing the involvement of electrophilic metallonitrene intermediate **18A**.

### Manganese-catalyzed late-stage C–H methylation

The incorporation of methyl groups has the potential to manipulate absorption, distribution, metabolism, and excretion (ADME), solubility, and protein–ligand binding properties as well as biological activities of small molecules, potentially leading to dramatic increases in potency, and thus has been widely explored in drug discovery [54–56]. Late-stage C–H methylation has recently been investigated using iron and cobalt catalysts as sustainable 3d metal catalysts [57–58], while manganese-catalyzed C–H methylations are scarce [59–60]. This disparity is due to the challenges in functional group tolerance of Mn-mediated late-stage transformations.

**Scheme 7:**
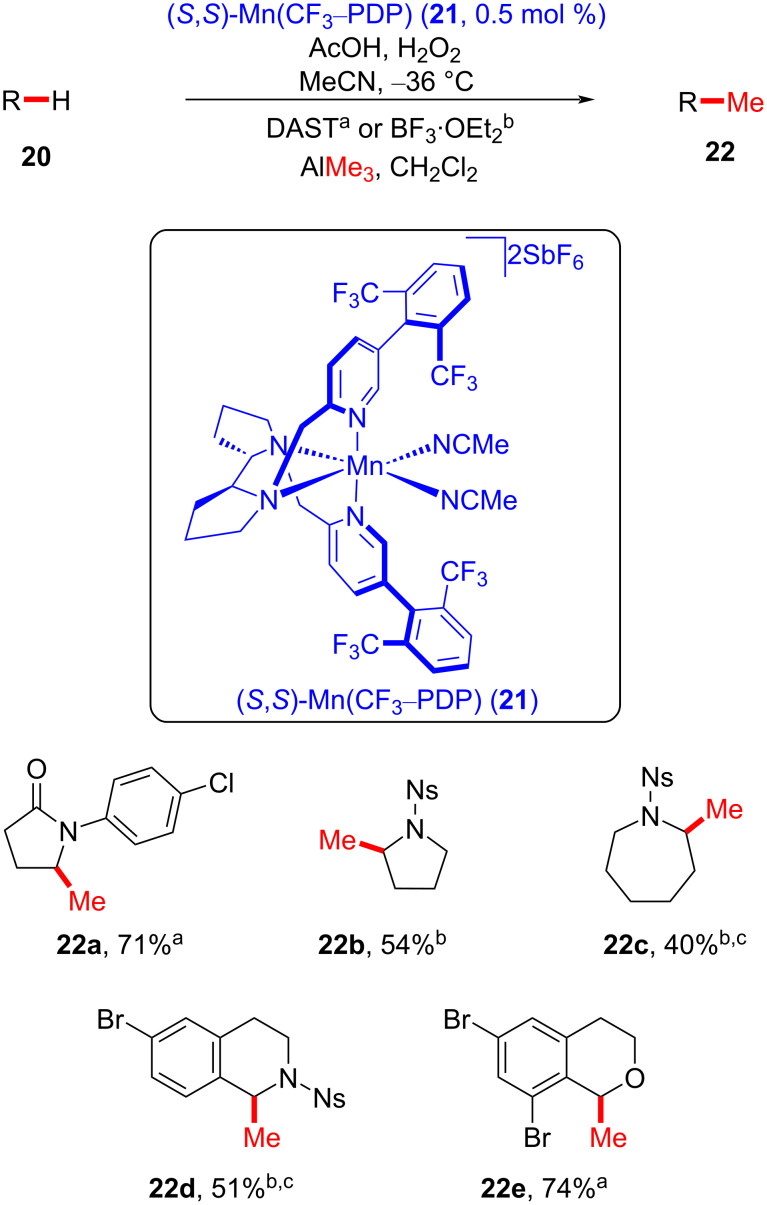
Mn-catalyzed C–H methylation of heterocyclic scaffolds commonly found in small-molecule drugs. ^a^DAST activation. ^b^BF_3_⋅OEt_2_ activation. ^c^1 mol % of (*S*,*S*)-Mn(CF_3_–PDP) was used.

The White group reported a late-stage Mn-catalyzed C–H methylation protocol that utilizes an external Lewis acid and trimethylaluminum as a methyl source (Scheme 7) [61]. The late-stage methylation of simple heterocyclic motifs was initially investigated using (*S*,*S*)-Mn(CF_3_–PDP) (**21**), providing methylated lactams **22a**–**e**. Notably, methylation site selectivity was observed for the carbon atoms adjacent to a heteroatom, such as a nitrogen (see **22a**–**d**) or oxygen (see **22e**).

**Scheme 8:**
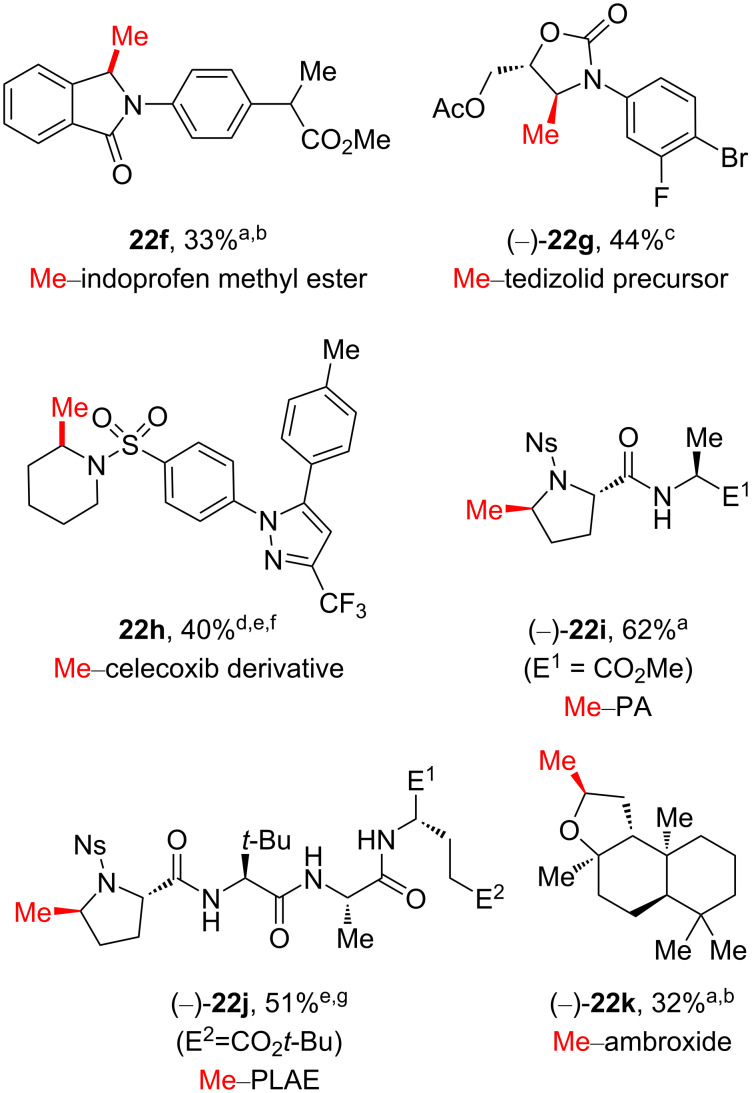
Examples of late-stage C–H methylation of bioactive molecules. ^a^DAST activation. ^b^For insoluble substrates, CH_2_Cl_2_ was added and/or the reaction temperature of the oxidation reaction was increased to 0 °C. ^c^TMSOTf activation. ^d^BF_3_⋅OEt_2_ activation. ^e^Oxidation intermediate was isolated before methylation. ^f^HBF_4_ protection was performed. ^g^Deoxo-Fluro^®^ activation.

Moreover, the above described manganese-catalyzed late-stage methylation process was implemented to provide methylated bioactive molecules, effectively avoiding conventionally lengthy de novo synthetic pathways (Scheme 8). The late-stage methylation of complex drug derivatives, such as of indoprofen (anti-inflammatory), tedizolid (antibiotic), and celecoxib (anti-inflammatory), successfully delivered methylated drug candidates **22f**–**h**, respectively. In addition, methylation of proline-containing multipeptides was achieved via fluorine activation (see **22i**–**j**), and the oxygen-containing natural terpenoid ambroxide was methylated at the methylene position next to the O atom on the tetrahydrofuran ring (see **22k**). This manganese-catalyzed late-stage approach enables the direct methylation of unactivated C–H bonds with excellent site selectivity, which is observed at the more electron-rich and the less sterically hindered position.

### Manganese-catalyzed late-stage C–H alkynylation

Alkynes are invaluable intermediates in organic synthesis and are conventionally prepared via palladium-catalyzed cross-coupling reactions [62]. Moreover, they constitute an important structural modality in small-molecule drugs, such as levonorgestrel (birth control drug), efavirenz (HIV/AIDS treatment), and erlotinib (anticancer). Although step-economical C–H alkynylations have been investigated with 4d and 5d transition metals, 3d metal-catalyzed late-stage C–H alkynylations of bioactive structural motifs are rare [63].

**Scheme 9:**
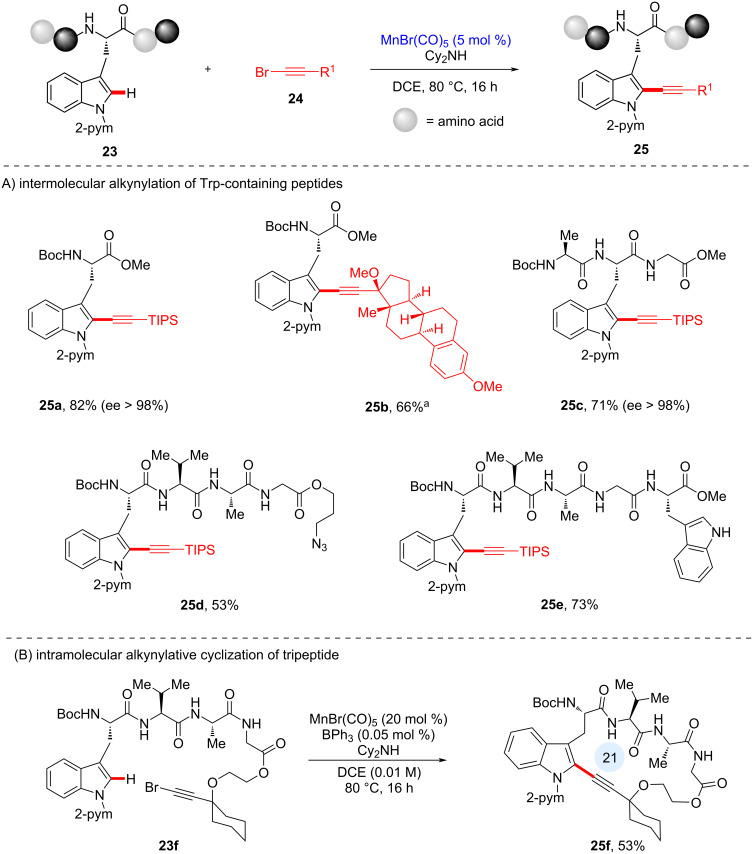
A) Mn-catalyzed late-stage C–H alkynylation of peptides. B) Intramolecular late-stage alkynylative cyclic peptide formation. ^a^0.05 mol % of BPh_3_ was added.

In 2017, the Ackermann group disclosed a late-stage Mn(I)-catalyzed C–H alkynylation of various complex peptide scaffolds [64]. As shown in Scheme 9, manganese(I) catalysis remarkably resulted in racemization-free alkynylation, representing a step-economical approach to several tryptophan-containing peptides with significant potential for drug discovery and medicinal chemistry. Positional selectivity was observed at the C2 position due to the presence of the pyrimidine directing group. Interestingly, alkynylative conjugation of tryptophan to a steroid motif was successfully achieved (see **25b**). In addition to accessing the monopeptide **25a**, tri-, tetra-, and pentapeptides **25c**–**e**, respectively, were obtained under the developed reaction conditions. It is noteworthy that substrates containing an azido group (see **25d**) or free NH (see **25e**) were tolerated, demonstrating significant bioorthogonality in manganese(I) catalysis. The robustness of the method bears significance for further synthetic applications, such as “Click” chemistry or N-functionalization. Moreover, as shown in Scheme 9B, the manganese(I) catalysis regime enabled peptide macrocyclization (see **25f**).

**Figure 7:**
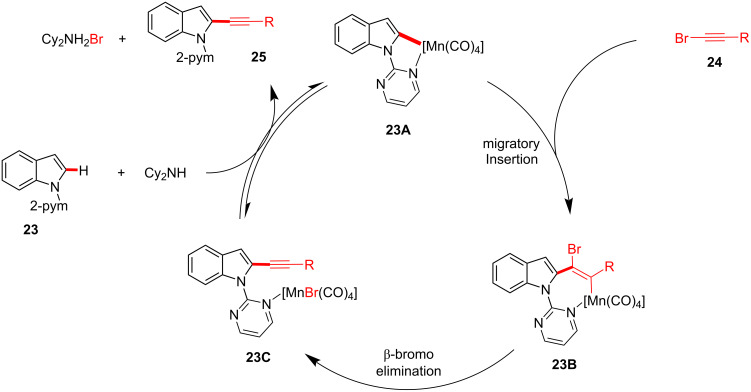
Proposed reaction mechanism of Mn(I)-catalyzed C–H alkynylation.

Based on additional mechanistic investigations, it was proposed that substrate **23** forms five-membered manganacycle complex **23A** under basic conditions, which undergoes alkyne insertion to provide seven-membered manganacycle complex **23B** (Figure 7). Subsequently, intermediate **23B** undergoes β-bromo elimination to produce **23C**, whereby the addition of BPh_3_ presumably accelerates this process [65]. Subsequently, the manganese species participate in the catalytic cycle by yielding alkynylated product **25**. However, the mechanism entailing oxidative addition, followed by reductive elimination could not be ruled out.

### Manganese-catalyzed late-stage C–H allylation

**Scheme 10:**
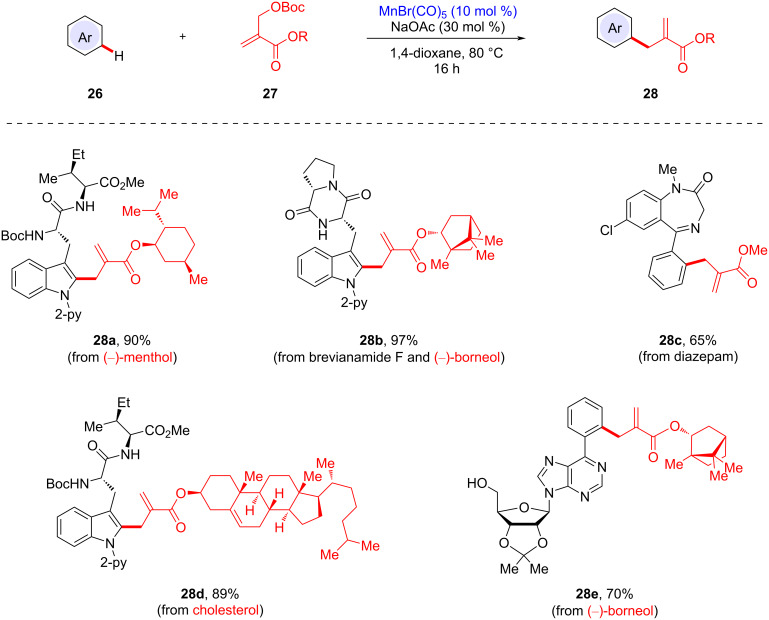
Late-stage Mn-catalyzed C–H allylation of peptides and bioactive motifs.

The late-stage modification of peptides has received increasing attention due to the convenient and efficient modality. However, such protocols generally require substrate prefunctionalization and expensive metal catalysts, such as Pd [66–87], Rh [88–91], and Ru [92–93]. In 2019, the Ackermann group demonstrated that a manganese(I) catalyst enabled the late-stage C–H allylation of structurally complex peptides in a site-selective fashion (Scheme 10) [94]. Based on an initial optimization study, manganese(I) pentacarbonyl bromide was deemed as the optimal catalyst, enabling a robust racemization-free allylation process. In addition to tryptophan-containing peptides, diazepam and nucleoside analogues were found to be viable allylation substrates, affording highly complex peptides.

**Scheme 11:**
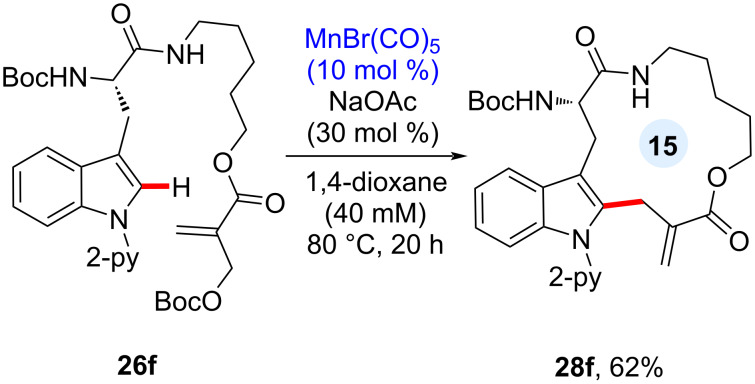
Intramolecular C–H allylative cyclic peptide formation.

Cyclic peptides are known to be structurally and chemically stable against enzymatic degradation because the cyclic skeleton restricts the conformation and limits β-turns. In this manganese catalysis, the late-stage C–H allylation manifold was extended to the construction of a cyclic peptide motif (Scheme 11). Dipeptide substrate **26f** decorated with a Morita–Baylis–Hillman carbonate underwent the intramolecular C–H allylation process to yield cyclic peptide **28f** under dilute reaction conditions. This macrocyclization strategy introduces an exocyclic olefin motif onto the cyclic peptide, which can be further utilized as a Michael acceptor for a variety of nucleophiles.

**Scheme 12:**
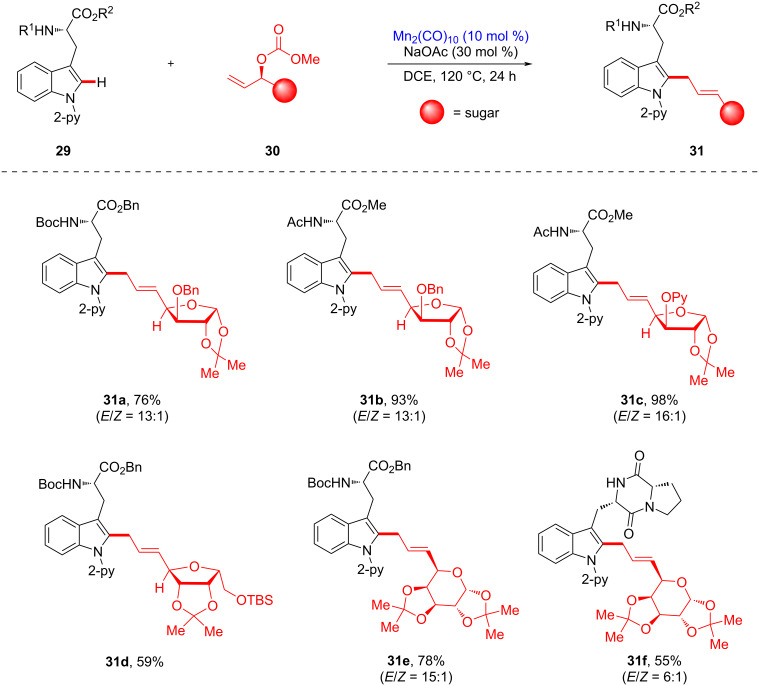
Late-stage C–H glycosylation of tryptophan analogues.

Based on their manganese-catalyzed allylation using Morita–Baylis–Hillman carbonates, the Ackermann group established an applicable late-stage C–H glycosylation of peptides (Scheme 12) [95]. Thus, allylative peptide–carbohydrate conjugation was achieved using tryptophan-containing peptides **29** and sugar-containing allyl carbonates **30** in chemo- and site-selective manners using a pyridyl directing group. The optimized reaction conditions entailed the use of dimanganese decacarbonyl as the catalyst and sodium acetate as the base to deliver the corresponding peptide–sugar conjugates **31** at the late stage. Notably, the chemoselective glycoconjugation strategy was compatible with various sugar scaffolds, affording glycotryptophans bearing either furanose or pyranose motifs. In addition, a brevianamide F analogue, a natural product scaffold, was transformed into glycosylated tryptophan **31f**. It is noteworthy that this manganese(I)-catalyzed glycoconjugation method avoids racemization.

**Scheme 13:**
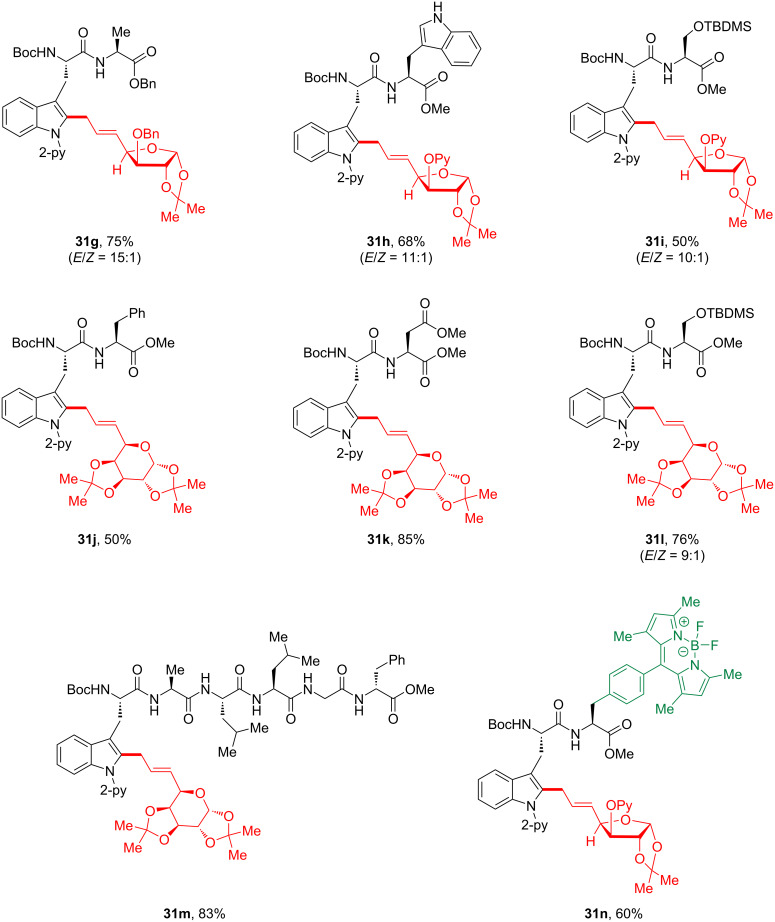
Late-stage C–H glycosylation of tryptophan-containing peptides.

Furthermore, manganese-catalyzed allylative linchpin C–H glycosylation was investigated using structurally sophisticated tryptophan-containing peptides (Scheme 13). A wide variety of complex peptides was explored, affording glycosylated conjugates with high stereoselectivity. The free NH functional group was tolerated in the manganese catalysis protocol, suggesting that the chemoselectivity was controlled by chelation of the adjacent directing group (see **31h**). This manganese(I)-catalyzed late-stage glycosylation provides hexaglycopeptide conjugate **31m** without epimerization. Moreover, the late-stage C–H diversification process enabled bioorthogonal access to glycosylated peptides, such as a fluorescent BODIPY-labeled tryptophan **31n**, regarded as a potentially viable peptide-based biosensor.

### Manganese-catalyzed inter- and intramolecular C–H alkenylations

Manganese(I)-catalyzed C–H alkenylation of 2-phenylpyridines or *N*-pyridinylindoles with alkynes is characterized by proximity-induced C–H activation through chelation assistance. However, application of this protocol for late-stage functionalization remains challenging [96–103].

**Scheme 14:**
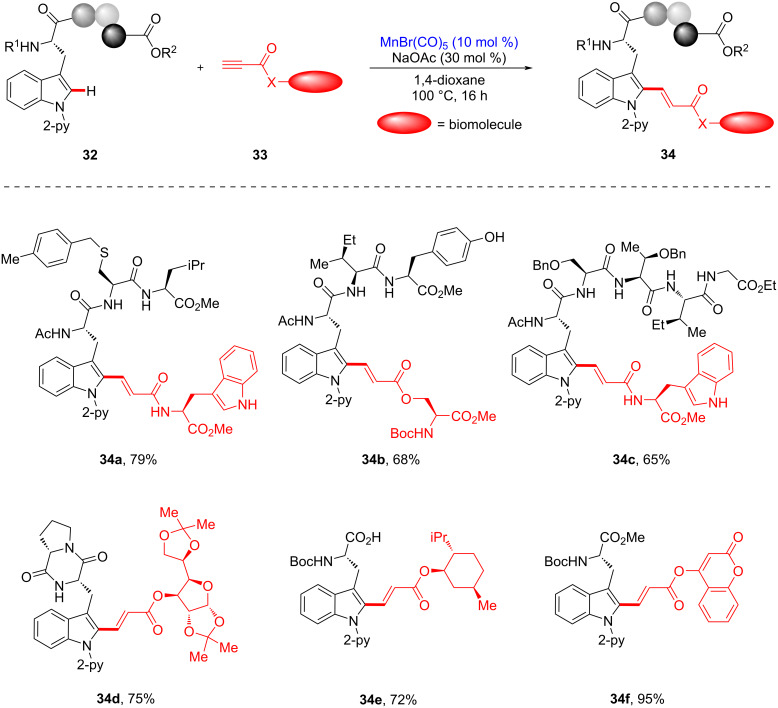
Late-stage C–H alkenylation of tryptophan-containing peptides.

In 2021, it was revealed by the Ackermann group that manganese(I) catalysis enabled the bioorthogonal late-stage alkenylation of structurally sophisticated peptides [104]. The manganese(I)-catalyzed intermolecular alkenylation of tryptophan-containing peptides **32** was performed under basic conditions, yielding hybrid peptides **34** without racemization, containing a *trans*-alkene linker bearing biologically active motifs in chemo- and site-selective manners assisted by the pyridyl directing group (Scheme 14). For example, a substrate containing a free OH or NH was successfully alkenylated at the late stage, suggesting a high functional group tolerance (see **34a** and **34b**). In addition, a more complex pentapeptide provided the corresponding product **34c**, bearing a free-NH tryptophan. Notably, alkenylative ligation of tryptophan-containing peptides and alkynes containing biomolecular motifs including sugar, menthol, or coumarin units was successful, delivering unprecedented hybrid complex peptides **34d**–**f** in good yield.

**Scheme 15:**
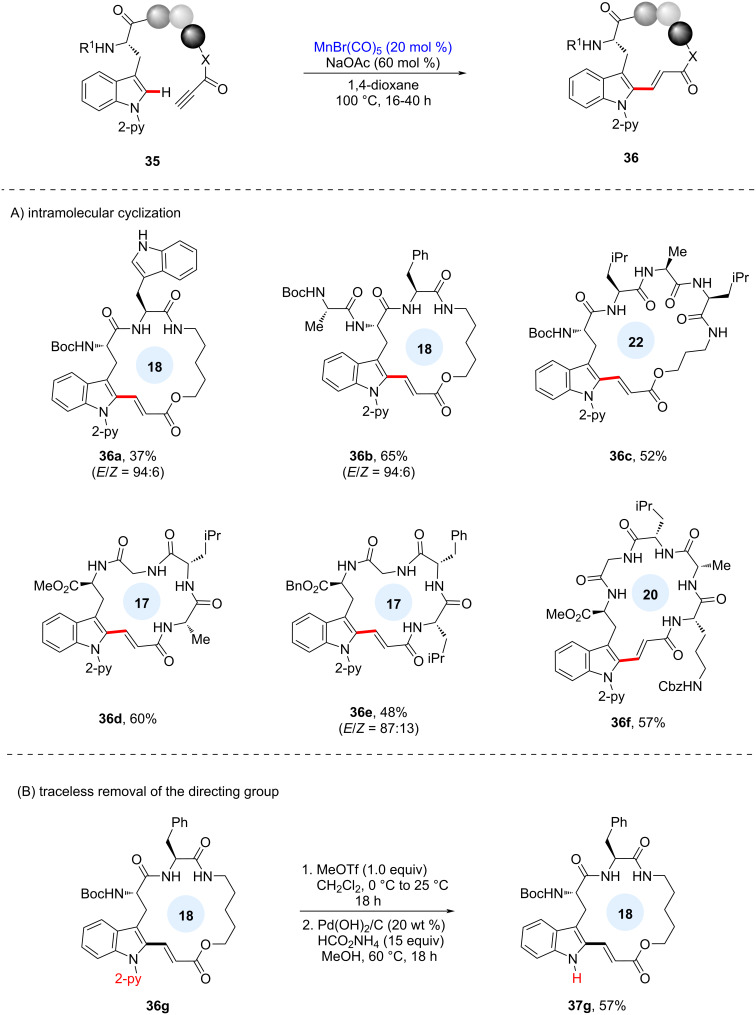
A) Late-stage C–H macrocyclization of tryptophan-containing peptides and B) traceless removal of pyridyl directing group.

Based on the developed intermolecular process, late-stage intramolecular C–H macrocyclization was also investigated (Scheme 15). To avoid unwanted intermolecular oligomerization, the reaction was performed at a high dilution, furnishing either C- or N-terminus-alkenylated products with excellent chemoselectivity. In this macrocyclization, cyclic multipeptides of varying ring size were successfully obtained with excellent functional group tolerance. In addition, selective *N*-methylation of the 2-pyridine directing group and successive hydrogenation processes provided an efficient traceless removal of the directing group, affording free-NH tryptophan-containing peptide **37g**.

## Conclusion

Metal-catalyzed late-stage functionalization has shown significant potential in the fields of medicinal chemistry, agrochemistry, and chemical biology. While transition metal catalysis has been a reliable and efficient strategy for late-stage functionalization, it suffers several disadvantages, such as the need for additional prefunctionalization and the use of expensive and toxic precious metals. To avoid these issues, 3d-metal-catalyzed C–H functionalization has recently been realized as a sustainable catalytic system and is actively being investigated for various late-state functionalization purposes. Notably, late-stage functionalization with manganese catalysts offers a sustainable catalytic system, and recent advancements have allowed for the construction of diversified bioactive small molecules and peptides through late-stage C–H aminations, azidations, fluorinations, allylations, alkynylations, alkenylations, and fluorescent labeling with BODIPY. Moreover, manganese catalysis exhibits excellent functional group tolerance in late-stage C–H functionalization, indicating a robust and versatile catalytic system. Several challenges still remain since there are multiple steps to prepare suitable high-valent Mn complexes. Furthermore, Mn(I)-catalyzed enantioselective C–H functionalization at the late stage is still underexplored. Given the sustainability and versatility of manganese-catalyzed late-stage functionalization, further advances are expected in the future, such as protecting-group-free methodologies, peptide biosensors, and facile functionalizations within unexplored realms of complex peptides.

## References

[1] Bruce M I, Iqbal M Z, Stone F G A (1970). J Chem Soc A.

[2] Carney J R, Dillon B R, Thomas S P (2016). Eur J Org Chem.

[3] Cano R, Mackey K, McGlacken G P (2018). Catal Sci Technol.

[4] Hu Y, Zhou B, Wang C (2018). Acc Chem Res.

[5] Gandeepan P, Müller T, Zell D, Cera G, Warratz S, Ackermann L (2019). Chem Rev.

[6] Aneeja T, Neetha M, Afsina C M A, Anilkumar G (2021). Catal Sci Technol.

[7] Lu X, He S-J, Cheng W-M, Shi J (2018). Chin Chem Lett.

[8] Wang W, Lorion M M, Shah J, Kapdi A R, Ackermann L (2018). Angew Chem, Int Ed.

[9] White M C, Zhao J (2018). J Am Chem Soc.

[10] Abrams D J, Provencher P A, Sorensen E J (2018). Chem Soc Rev.

[11] Baudoin O (2020). Angew Chem, Int Ed.

[12] Kelly C B, Padilla-Salinas R (2020). Chem Sci.

[13] Wu J (2014). Tetrahedron Lett.

[14] Yang X, Wu T, Phipps R J, Toste F D (2015). Chem Rev.

[15] Li X, Shi X, Li X, Shi D (2019). Beilstein J Org Chem.

[16] Szpera R, Moseley D F J, Smith L B, Sterling A J, Gouverneur V (2019). Angew Chem, Int Ed.

[17] Gillis E P, Eastman K J, Hill M D, Donnelly D J, Meanwell N A (2015). J Med Chem.

[18] Wang J, Sánchez-Roselló M, Aceña J L, del Pozo C, Sorochinsky A E, Fustero S, Soloshonok V A, Liu H (2014). Chem Rev.

[19] Purser S, Moore P R, Swallow S, Gouverneur V (2008). Chem Soc Rev.

[20] Smart B E (2001). J Fluorine Chem.

[21] Meanwell N A (2018). J Med Chem.

[22] Liu W, Huang X, Cheng M-J, Nielsen R J, Goddard W A, Groves J T (2012). Science.

[23] Oh S, Jeong I H, Shin W-S, Wang Q, Lee S (2006). Bioorg Med Chem Lett.

[24] Liu W, Groves J T (2010). J Am Chem Soc.

[25] Huang X, Liu W, Ren H, Neelamegam R, Hooker J M, Groves J T (2014). J Am Chem Soc.

[26] Miller P W, Long N J, Vilar R, Gee A D (2008). Angew Chem, Int Ed.

[27] Tredwell M, Gouverneur V (2012). Angew Chem, Int Ed.

[28] Jacobson O, Kiesewetter D O, Chen X (2015). Bioconjugate Chem.

[29] Preshlock S, Tredwell M, Gouverneur V (2016). Chem Rev.

[30] Kolb H C, Finn M G, Sharpless K B (2001). Angew Chem, Int Ed.

[31] Moses J E, Moorhouse A D (2007). Chem Soc Rev.

[32] Hein C D, Liu X-M, Wang D (2008). Pharm Res.

[33] Jewett J C, Bertozzi C R (2010). Chem Soc Rev.

[34] Liang L, Astruc D (2011). Coord Chem Rev.

[35] Thirumurugan P, Matosiuk D, Jozwiak K (2013). Chem Rev.

[36] Tang W, Becker M L (2014). Chem Soc Rev.

[37] Poonthiyil V, Lindhorst T K, Golovko V B, Fairbanks A J (2018). Beilstein J Org Chem.

[38] Huang X, Bergsten T M, Groves J T (2015). J Am Chem Soc.

[39] Silverman R B (2008). Angew Chem, Int Ed.

[40] Niu L, Jiang C, Liang Y, Liu D, Bu F, Shi R, Chen H, Chowdhury A D, Lei A (2020). J Am Chem Soc.

[41] Wenk G L, Danysz W, Mobley S L (1995). Eur J Pharmacol, Environ Toxicol Pharmacol Sect.

[42] Gideons E S, Kavalali E T, Monteggia L M (2014). Proc Natl Acad Sci U S A.

[43] Meyer T H, Samanta R C, Del Vecchio A, Ackermann L (2021). Chem Sci.

[44] Huang X, Zhuang T, Kates P A, Gao H, Chen X, Groves J T (2017). J Am Chem Soc.

[45] Mayer J M (2011). Acc Chem Res.

[46] Capaldo L, Ravelli D (2017). Eur J Org Chem.

[47] Richter M F, Drown B S, Riley A P, Garcia A, Shirai T, Svec R L, Hergenrother P J (2017). Nature.

[48] Campoli-Richards D M, Brogden R N (1987). Drugs.

[49] Park Y, Kim Y, Chang S (2017). Chem Rev.

[50] Paradine S M, White M C (2012). J Am Chem Soc.

[51] Hennessy E T, Betley T A (2013). Science.

[52] Paradine S M, Griffin J R, Zhao J, Petronico A L, Miller S M, Christina White M (2015). Nat Chem.

[53] Clark J R, Feng K, Sookezian A, White M C (2018). Nat Chem.

[54] Barreiro E J, Kümmerle A E, Fraga C A M (2011). Chem Rev.

[55] Leung C S, Leung S S F, Tirado-Rives J, Jorgensen W L (2012). J Med Chem.

[56] Schönherr H, Cernak T (2013). Angew Chem, Int Ed.

[57] Zhu N, Zhao J, Bao H (2017). Chem Sci.

[58] Friis S D, Johansson M J, Ackermann L (2020). Nat Chem.

[59] Sato T, Yoshida T, Al Mamari H H, Ilies L, Nakamura E (2017). Org Lett.

[60] Liu W, Cera G, Oliveira J C A, Shen Z, Ackermann L (2017). Chem – Eur J.

[61] Feng K, Quevedo R E, Kohrt J T, Oderinde M S, Reilly U, White M C (2020). Nature.

[62] Sonogashira K, Tohda Y, Hagihara N (1975). Tetrahedron Lett.

[63] Caspers L D, Nachtsheim B J (2018). Chem – Asian J.

[64] Ruan Z, Sauermann N, Manoni E, Ackermann L (2017). Angew Chem, Int Ed.

[65] Wierschke S G, Chandrasekhar J, Jorgensen W L (1985). J Am Chem Soc.

[66] Gong W, Zhang G, Liu T, Giri R, Yu J-Q (2014). J Am Chem Soc.

[67] Vinogradova E V, Zhang C, Spokoyny A M, Pentelute B L, Buchwald S L (2015). Nature.

[68] Zhu Y, Bauer M, Ackermann L (2015). Chem – Eur J.

[69] Mondal B, Roy B, Kazmaier U (2016). J Org Chem.

[70] Lee H G, Lautrette G, Pentelute B L, Buchwald S L (2017). Angew Chem, Int Ed.

[71] Liu T, Qiao J X, Poss M A, Yu J-Q (2017). Angew Chem, Int Ed.

[72] Noisier A F M, García J, Ionuţ I A, Albericio F (2017). Angew Chem, Int Ed.

[73] Rojas A J, Zhang C, Vinogradova E V, Buchwald N H, Reilly J, Pentelute B L, Buchwald S L (2017). Chem Sci.

[74] Tang J, He Y, Chen H, Sheng W, Wang H (2017). Chem Sci.

[75] Bauer M, Wang W, Lorion M M, Dong C, Ackermann L (2018). Angew Chem, Int Ed.

[76] Kubota K, Dai P, Pentelute B L, Buchwald S L (2018). J Am Chem Soc.

[77] Wang W, Lorion M M, Martinazzoli O, Ackermann L (2018). Angew Chem, Int Ed.

[78] Zhan B-B, Li Y, Xu J-W, Nie X-L, Fan J, Jin L, Shi B-F (2018). Angew Chem, Int Ed.

[79] Zhang X, Lu G, Sun M, Mahankali M, Ma Y, Zhang M, Hua W, Hu Y, Wang Q, Chen J (2018). Nat Chem.

[80] Bai Q, Tang J, Wang H (2019). Org Lett.

[81] Li B, Li X, Han B, Chen Z, Zhang X, He G, Chen G (2019). J Am Chem Soc.

[82] Yuan F, Hou Z-L, Pramanick P K, Yao B (2019). Org Lett.

[83] Zhan B-B, Fan J, Jin L, Shi B-F (2019). ACS Catal.

[84] Liu L, Liu Y-H, Shi B-F (2020). Chem Sci.

[85] Weng Y, Ding X, Oliveira J C A, Xu X, Kaplaneris N, Zhu M, Chen H, Chen Z, Ackermann L (2020). Chem Sci.

[86] Wu J, Kaplaneris N, Ni S, Kaltenhäuser F, Ackermann L (2020). Chem Sci.

[87] Zhan B-B, Jiang M-X, Shi B-F (2020). Chem Commun.

[88] Key H M, Miller S J (2017). J Am Chem Soc.

[89] Liu J, Liu X, Zhang F, Qu J, Sun H, Zhu Q (2020). Chem – Eur J.

[90] Peng J, Li C, Khamrakulov M, Wang J, Liu H (2020). Org Lett.

[91] Wang W, Wu J, Kuniyil R, Kopp A, Lima R N, Ackermann L (2020). Chem.

[92] Schischko A, Ren H, Kaplaneris N, Ackermann L (2017). Angew Chem, Int Ed.

[93] Schischko A, Kaplaneris N, Rogge T, Sirvinskaite G, Son J, Ackermann L (2019). Nat Commun.

[94] Kaplaneris N, Rogge T, Yin R, Wang H, Sirvinskaite G, Ackermann L (2019). Angew Chem, Int Ed.

[95] Wang W, Subramanian P, Martinazzoli O, Wu J, Ackermann L (2019). Chem – Eur J.

[96] Zhou B, Chen H, Wang C (2013). J Am Chem Soc.

[97] Shi L, Zhong X, She H, Lei Z, Li F (2015). Chem Commun.

[98] Wang H, Pesciaioli F, Oliveira J C A, Warratz S, Ackermann L (2017). Angew Chem, Int Ed.

[99] Zell D, Dhawa U, Müller V, Bursch M, Grimme S, Ackermann L (2017). ACS Catal.

[100] Wang C, Rueping M (2018). ChemCatChem.

[101] Ma X, Dang Y (2019). J Org Chem.

[102] Cembellín S, Dalton T, Pinkert T, Schäfers F, Glorius F (2020). ACS Catal.

[103] Wan S, Luo Z, Xu X, Yu H, Li J, Pan Y, Zhang X, Xu L, Cao R (2021). Adv Synth Catal.

[104] Kaplaneris N, Kaltenhӓuser F, Sirvinskaite G, Fan S, De Oliveira T, Conradi L-C, Ackermann L (2021). Sci Adv.

